# Isolated hepatic actinomycosis: a case report

**DOI:** 10.1186/1752-1947-4-45

**Published:** 2010-02-08

**Authors:** Thomas Lall, Thomas M Shehab, Paul Valenstein

**Affiliations:** 1 Department of Internal Medicine, Saint Joseph Mercy Hospital, 5301 McAuley Drive, Ypsilanti, Michigan, MI 48197, USA; 2 Huron Gastroenterology, 5300 Elliott Drive, Ypsilanti, MI 48197, USA; 3 Department of Pathology, Saint Joseph Mercy Hospital, 5301 McAuley Drive, Ypsilanti, Michigan, MI 48197, USA

## Abstract

**Introduction:**

Actinomyces are slow growing, non-spore forming, gram-positive, branching bacilli that thrive in anaerobic and microareophilic conditions. Actinomyces are more commonly associated with oral and cervicofacial infections. Hepatic involvement in infections of the abdomen (known as isolated hepatic actinomycosis) is rare, accounting for only 5% of all cases of actinomycosis.

**Case presentation:**

We present the case of a 75-year-old Caucasian woman with a 3-month history of night sweats, fever, chills, abdominal bloating, anorexia, weight-loss, and early satiety. The patient was found to have isolated hepatic actinomycosis infection after undergoing a laparotomy with a biopsy of the liver. The patient has now recovered.

**Conclusion:**

Isolated hepatic actinomycosis is a rare and often overlooked etiology for a liver mass. Given its subacute presentation and nondescript symptomatology, physicians should be aware of this differential and the potential pitfalls in diagnosis and management.

## Introduction

Actinomyces are slow growing, non-spore forming, gram-positive, branching bacilli that thrive in anaerobic and microareophilic conditions. It is most commonly associated with infection of the oral and cervicofacial region. Abdominal actinomycosis is rare and its pathogenesis is presumed to be hematogenous spread through the portal vein from a mucosal injury or other abdominal focus of infection. Most frequently affected is the ileocecal region. Hepatic involvement has been reported in 15% of those with abdominal infections, and represents 5% of all cases of actinomycosis [1]. Diagnosis is often difficult because the predominant presenting symptoms of fever, abdominal pain, and weight loss are non-specific. Imaging is many times suspicious for neoplasm and positive cultures are notoriously difficult to obtain, making the preoperative rate of diagnosis less than 10%.

In this case report, we report a case of isolated hepatic actinomycosis (IHA) occurring in a 75-year-old female with no predisposing factors. Along with a review of the literature, we will define the risk factors, clinical characteristics, diagnostic methods, and treatment of this infection.

## Case presentation

A 75-year-old Caucasian woman of northern European descent presented with a 3-month history of night sweats, fevers (101-102°F nightly), chills, abdominal bloating, anorexia with 60 lb weight loss and early satiety. Her past medical history included hypertension, hypothyroidism, gastroesophageal reflux disease, diverticulitis, polymyalgia rheumatica, and a laparoscopic cholecystectomy in 1997. Current medications included HydroDIURIL, Toprol XL, Diovan, Synthroid, Prilosec, Plaquinil, and calcium supplementation. She denied any abdominal pain, nausea, vomiting, diarrhea or constipation and was able to tolerate per os diet despite her early satiety.

Physical examination revealed an elderly female who did not appear to be in any acute distress. Vital signs: temperature 100.7°F, pulse 54/bpm, respirations 20/min, blood pressure 116/56 mm/Hg, oxygen saturation 98% on room air. Abdominal exam revealed no palpable hepatosplenomegaly, guarding or rebound with normoactive bowel sounds. The rest of her exam was unremarkable. Laboratory studies demonstrated a leukocytosis of 12.8 10^3^/mcL (4.0-10.0) and an absolute neutrophil count of 10.9 10^3^/mcL (1.7-1.6). Hemoglobin level, hematocrit, platelet count and mean corpuscular volume were 8.2 gm/dL (12.0-16.0), 23.9% (36-48), 341 10^3^/mcL (140-450) and 85 FL (82-100) respectively. The following liver chemistries were normal; serum glutamic oxaloacetic transaminase 17 IU/L (15-41), serum glutamic pyruvic transaminase 15 IU/L (10-15), total bilirubin 0.6 mg/dL (0.0-2.0) and direct bilirubin 0.2 mg/dL (0.0-0.8). Prothrombin time and international normalized ratio were 14.9 sec (8.0-12.5) and 1.30 respectively. There was an increase in alkaline phosphotase to 270 IU/L (27-120), tumor marker CA19-9 was 49.6 U/mL (0-35) and alpha fetoprotein was elevated to a level of 7.3 IU/mL (< 5.5). Albumin was low at a level of 1.8 gm/dL (3.2-4.8) as was serum protein 5.0 gm/dL (6.1-7.6). C-reactive protein, a marker of inflammation, was elevated to a level of 10.8 mg/dL (< 0.5).

**Figure 1:**
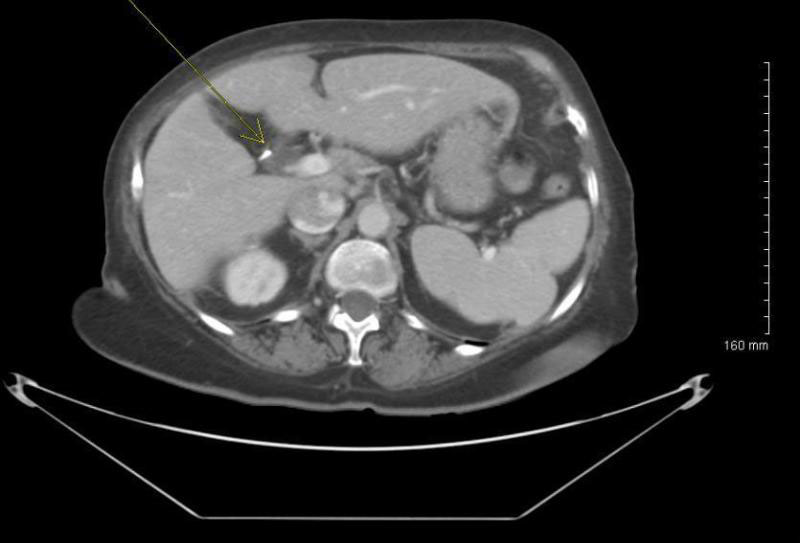
**CT scan of the liver demonstrating mass concerning for abscess and tumor**.

**Figure 2:**
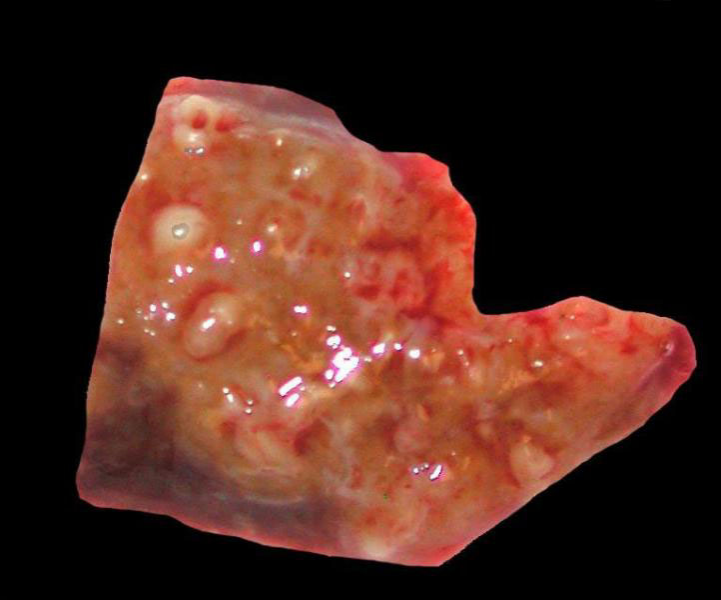
**Grosse liver biopsy demonstrating multiple micro-abscesses**.

**Figure 3:**
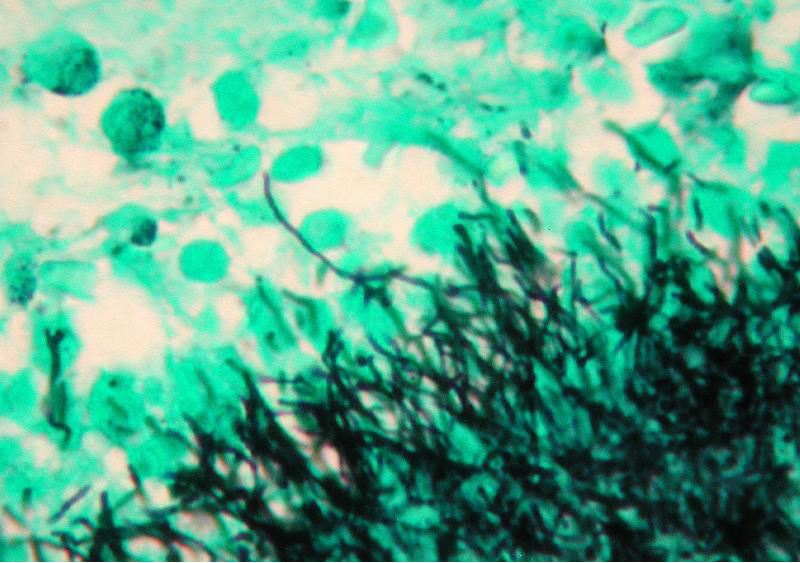
**Tissue gram-stain demonstrating elongated, gram-positive branching bacilli**.

**Figure 4:**
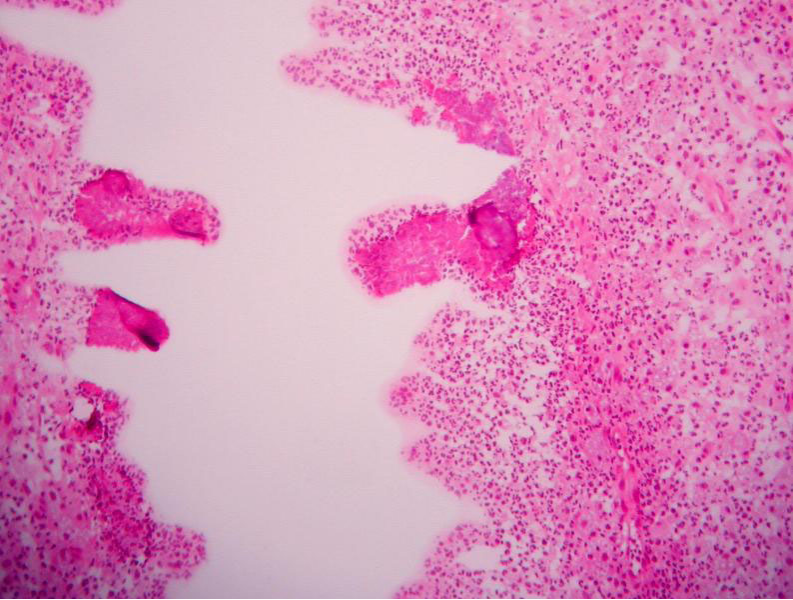
**Frozen section demonstrating the characteristic sulfur granules**.

The patient underwent imaging with an ultrasound of the liver and a CT of the abdomen. Both demonstrated a lesion in the posterior right lobe of the liver near the dome, measuring 6.9 × 7.4 cm, concerning for abscess and malignancy (Figure 1). This area was further characterized as not appearing fluid-like nor did it contain gas that would have allowed for confirmation of abscess. The patient was started on ciprofloxacin and flagyl for treatment of a potential abscess. Neoplasia remained a concern at this point, as several 1 cm. nodules were visualized at the lung bases and were concerning for metastatic disease. Gastroenterology, as well as infectious disease consults were obtained and the patient subsequently underwent liver core biopsy and aspiration. These tests revealed normal hepatocytes and organizing abscess respectively and both were negative for neoplasm. Unfortunately the specimens recovered from the core biopsy and aspiration were not stained for actinomycetes or sulfur granules, which could have lead to earlier diagnosis. Taking into consideration the patient's presenting symptoms, multiple lung nodules and lack of positive tissue diagnosis, malignancy could not be ruled out. For definitive diagnosis the patient was to undergo a video-assisted thoracic surgical (VATS) lung biopsy to obtain frozen section for culture and histochemical staining. The lung biopsy revealed fibrotic granulomata and were non-diagnostic for malignancy. A general surgeon then performed a laparotomy with biopsy of the liver. The liver specimen revealed neutrophilic microabscesses (Figure 2) containing gram-positive, filamentous, radially oriented bacteria in Brown and Brenn stained sections, consistent with actinomycosis (Figure 3). Characteristic sulfur granules were demonstrated on frozen section as well (Figure 4). The patient tolerated these procedures well and continued on a course of intravenous clindamycin for 15 days with transition to oral dosing lasting for a total of 6 months. She fully recovered and later imaging with CT demonstrated complete resolution of the liver abnormality.

## Discussion

Actinomyces is a rare cause of intra-abdominal infections. The etiology in the majority of cases involving the liver, are thought to arise directly or hematogenically from an intra-abdominal focus. In rare cases that involve the liver, when a primary focus is not found, the disease process is called primary or isolated hepatic actinomycosis (IHA). There are 13 different species of actinomycosis; 6 of which are associated with human disease (israelii, naeslundii, bovis, odontolyticus, viscosus and arachnia propionica). By far the most common human pathogen encountered is *Actinomyces israelii*, giving rise to chronic suppurative infections [2,3].

Risk factors include previous abdominal/pelvic surgery, abdominal wall trauma, gastrointestinal foreign body, gastrointestinal tract lesions and immunosupression [2]. Although the cause in the majority of cases is unknown (80.7%), the abscess can appear after any disruption in mucosal integrity [2]. Recent literature has cited an increased risk for this infection with biliary and pancreatic stent placement [4]. Association with the use of intrauterine contraceptive devices (IUD) is well documented as a cause of cervical-pelvic infection that can predispose to hematogenous spread [4,5]. Cases associated with abdominal or pelvic procedures were described in 17.5% of subjects reviewed in one study and were followed by infection at <1 year [6]. This would indicate that remote surgery (>1 yr previous) is not a risk factor for development of IHA. In our patient she had previously undergone a laparoscopic cholecystectomy >15 years prior to presentation. It is thought that this did not play a role in the development of her infection. Despite our case involving an elderly female, there is a male predominance for hepatic actinomycotic abscess (70-97%) with 30-50 years being the most common age group [4,6]. One potential cause for concern in our patient was her use of the immunosuppressive medication Plaquinil, which interferes with antigen presentation to host immune cells. It is possible that the use of this medication predisposed her to opportunistic infection.

Due to the cryptogenic nature of the disease, it takes several weeks to months of symptoms to make the diagnosis with a range of 4 days to 18 months until presentation [5]. The most common symptoms are non-specific, including fever (83.3%), abdominal pain (74.5%) and weight loss (50.9%) [7]. Laboratory examination often demonstrates a leukocytosis (75%) with left shift and elevation of alkaline phosphatase (83.3%), as seen in our patient [2,6]. Several studies have also shown an association with elevated CA19-9 levels. CA19-9 is a carbohydrate epitope that is sialylated on the surface of certain tumors. It is not organ specific and is clinically used as a marker of pancreatic, hepatobiliary and gastric malignancies [8]. This marker is often elevated in benign disease states of the hepatobiliary system, renal failure, pleural effusion, intestinal pneumonia and systemic lupus erythematosus. With these types of benign conditions the elevation of CA19-9 is often lower than malignant conditions. At a level of 1000 U/ml the marker's positive predictive value approaches 100% [8]. In our patient the CA19-9 level was 49.6 U/ml, which may have been an indication that her presenting symptoms were not secondary to a malignant process.

Because imaging studies frequently reveal single or multiple lesions, actinomycosis is often misdiagnosed as a primary or metastatic tumor. The most frequent radiographical finding is a single hypodense mass/abscess (68.4%) [7]. CT often demonstrates multiloculated spaces occupying hypoattenuated lesions making a differential diagnosis of echinococcosis, amoebiasis, multiple pyogenic abscess and cystic neoplasm [4]. MRI is characterized by low-signal intensity on T1 and high-signal intensity on T2 weighted images [9].

Definitive diagnosis is based on histochemical, macroscopic, and microscopic examination of surgical tissue specimens, which reveal yellow sulfur granules and basophilic filament aggregates respectively [2]. Cultures can be positive but take up to 2 weeks to grow, secondary to low concentration and the propensity to grow slowly. One study demonstrated that blood culture was only positive in 2/13 (15.4%) cases reviewed [3,6]. Further complicating culture interpretation is that specimens are commonly mixed with other organisms (35.2%) [6]. Often the diagnosis requires surgical intervention to obtain specimens adequate to demonstrate the characteristic findings of sulfur granules and filament aggregates. Sharma et al. reviewed 35 cases in which 29 (82.9%) required surgical intervention to obtain specimens for diagnosis [6].

Both surgical excision and antimicrobial treatment are therapeutically effective. The medical treatment regimen for IHA is the administration of penicillin, tetracycline, or clindamycin [2]. Duration of treatment is variable with courses lasting from 3-6 months [4,5]. If antibiotics do not lead to cure, surgical excision of the mass is required for complete resolution as well as to rule out neoplasm. A review of the literature by Wong et al. showed that in one series, 28 of 53 cases were managed with antibiotics alone and only two of these required surgical intervention. They also reported no significant difference in mortality between those that received antibiotics alone and those that had surgical intervention in addition to antibiotics [10]. Recommended are follow up examinations with imaging studies such as ultrasound or CT to aid with duration of therapy.

## Conclusion

In conclusion, IHA is a rare and often overlooked etiology for a liver mass. Given its nonspecific presentation and nondescript symptomatology, physicians should be aware of this differential and the potential pitfalls in diagnosis and management.

## Abbreviations

IHA: Isolated hepatic actinomycosis; IUD: intrauterine contraceptive devices; VATS: video-assisted thoracic surgery.

## Competing interests

The authors declare that they have no competing interests.

## Authors' contributions

All authors contributed in the final approval of the article and to each of the following jobs: drafting of the article, critical revision of the article for important intellectual content, conception and design, analysis and interpretation of the data.

## Consent

Written informed consent was obtained from the patient for publication of this case report and accompanying images. A copy of the written consent is available for review by the Editor-in-Chief of this journal.
